# Low Complexity of Infection Is Associated With Molecular Persistence of *Plasmodium falciparum* in Kenya and Tanzania

**DOI:** 10.3389/fepid.2022.852237

**Published:** 2022-06-06

**Authors:** Hillary M. Topazian, Kara A. Moser, Billy Ngasala, Peter O. Oluoch, Catherine S. Forconi, Lwidiko E. Mhamilawa, Ozkan Aydemir, Oksana Kharabora, Molly Deutsch-Feldman, Andrew F. Read, Madeline Denton, Antonio Lorenzo, Nicole Mideo, Bernhards Ogutu, Ann M. Moormann, Andreas Mårtensson, Boaz Odwar, Jeffrey A. Bailey, Hoseah Akala, John Michael Ong'echa, Jonathan J. Juliano

**Affiliations:** ^1^Department of Infectious Disease Epidemiology, Imperial College, London, United Kingdom; ^2^Institute for Global Health and Infectious Diseases, University of North Carolina, Chapel Hill, NC, United States; ^3^Department of Parasitology and Medical Entomology, Muhimbili University of Health and Allied Sciences, Dar es Salaam, Tanzania; ^4^Department of Medicine, University of Massachusetts Chan Medical School, Worcester, MA, United States; ^5^Center for Global Health Research, Kenyan Medical Research Institute, Kisumu, Kenya; ^6^Department of Women's and Children's Health, International Maternal and Child Health, Uppsala University, Uppsala, Sweden; ^7^Department of Pathology and Laboratory Medicine, Brown University, Providence, RI, United States; ^8^Department of Epidemiology, Gillings School of Global Public Health, Chapel Hill, NC, United States; ^9^Department of Entomology, Penn State University, University Park, PA, United States; ^10^Department of Ecology and Evolutionary Biology, University of Toronto, Toronto, ON, Canada; ^11^Division of Infectious Diseases, School of Medicine, University of North Carolina, Chapel Hill, NC, United States; ^12^Curriculum in Genetics and Molecular Biology, School of Medicine, University of North Carolina, Chapel Hill, NC, United States

**Keywords:** *Plasmodium*, artemisinin, parasite clearance, Kenya, Tanzania, *P. falciparum*, artemisinin combination therapy

## Abstract

**Background:**

*Plasmodium falciparum* resistance to artemisinin-based combination therapies (ACTs) is a threat to malaria elimination. ACT-resistance in Asia raises concerns for emergence of resistance in Africa. While most data show high efficacy of ACT regimens in Africa, there have been reports describing declining efficacy, as measured by both clinical failure and prolonged parasite clearance times.

**Methods:**

Three hundred children aged 2–10 years with uncomplicated *P. falciparum* infection were enrolled in Kenya and Tanzania after receiving treatment with artemether-lumefantrine. Blood samples were taken at 0, 24, 48, and 72 h, and weekly thereafter until 28 days post-treatment. Parasite and host genetics were assessed, as well as clinical, behavioral, and environmental characteristics, and host anti-malarial serologic response.

**Results:**

While there was a broad range of clearance rates at both sites, 85% and 96% of Kenyan and Tanzanian samples, respectively, were qPCR-positive but microscopy-negative at 72 h post-treatment. A greater complexity of infection (COI) was negatively associated with qPCR-detectable parasitemia at 72 h (OR: 0.70, 95% CI: 0.53–0.94), and a greater baseline parasitemia was marginally associated with qPCR-detectable parasitemia (1,000 parasites/uL change, OR: 1.02, 95% CI: 1.01–1.03). Demographic, serological, and host genotyping characteristics showed no association with qPCR-detectable parasitemia at 72 h. Parasite haplotype-specific clearance slopes were grouped around the mean with no association detected between specific haplotypes and slower clearance rates.

**Conclusions:**

Identifying risk factors for slow clearing *P. falciparum* infections, such as COI, are essential for ongoing surveillance of ACT treatment failure in Kenya, Tanzania, and more broadly in sub-Saharan Africa.

## Introduction

*Plasmodium falciparum* resistance to first-line antimalarials remains a major threat to global malaria elimination efforts. Currently, artemisinin combination therapies (ACTs) are the cornerstone of antimalarial chemotherapy and are considered the first-line treatment option for uncomplicated malaria worldwide (1). *Plasmodium falciparum* resistance to ACTs is defined by a delayed clearance phenotype in which parasites are cleared from the bloodstream at a slower rate than expected, as measured by microscopy positivity 3 days (72 h) after treatment initiation or by a prolonged parasite clearance half-life (2, 3). Drug-resistant parasites can heighten the duration, density, and infectivity of gametocytes to mosquitoes (4) and continue to be detrimental to human health through recrudescent infection and intensified parasite transmission.

The delayed clearance phenotype was first reported on the Cambodia/Thai border (5), and subsequently associated with the discovery of novel variants in a kelch gene on chromosome 13 (K13 mutations) (6). *Plasmodium falciparum* has evolved resistance to nearly every antimalarial drug in use (7, 8), and a pressing concern is that resistance to ACTs will spread to or independently evolve in sub-Saharan Africa, where the majority of the world's malaria deaths occur. Clinical trial data remain mixed; most trials testing the efficacy of ACTs across the African continent have shown no evidence of treatment failure (9–12), yet a small number of recent studies have reported efficacies below 90% (13, 14). Recent data around prolonged microscopic parasite clearance and the R561H K13 mutation in Rwanda, and the emergence of resistance in Uganda are of particular concern (15, 16). Additionally, several studies have reported reduced treatment efficacy to lumefantrine *in vitro*, a common partner drug choice for ACTs (17). Because of the potential for emerging resistance, the WHO recommends regular studies to assess the therapeutic efficacy of ACTs, including *in vitro* studies of parasite susceptibility to antimalarials in culture and frequent *in vivo* measurements of parasite clearance in representative patient populations (1). Slow clearance has rarely been observed through use of microscopy in Africa (18), however, molecular evidence of persistent infection has been frequently reported (19, 20). The clinical importance of persistence by qPCR remains unclear due to concerns that this may represent circulation of parasite DNA rather than true infection, but recent evidence has linked it to clinical failure (21).

There is evidence that there may be a broad range of parasite clearance rates among African patients likely driven by genetic diversity and greater complexity of infection (22). Such variation in clearance may complicate efforts to disentangle the effects of K13 or other mutations on ACT resistance. Host factors, from genetic to environmental characteristics, may also impact parasite clearance. Results from Southeast Asia and Kenya suggest a role of the host immune system in slow clearers (23, 24). Host genotype may also play a role in parasite clearance despite the presence of drug resistant parasite genotypes (25). Other factors, such as socioeconomic status, risk of malaria infections, and other environmental factors may also contribute to clearance times in ways we do not understand (26–28). Shedding light on risk factors for slow-clearing infection is critical to prepare for the onset of full-fledged ACT resistance.

Finally, transmission dynamic differences in sub-Saharan Africa may make ACT resistance manifest differently than in Southeast Asia. Sub-Saharan Africa comprises relatively high transmission areas compared to Southeast Asia, and therefore individuals are often infected with polyclonal infections made up of more than one genetically distinct parasite strain. Within-host dynamics could play a role in how ACT resistant parasites are maintained and selected for over the course of treatment (29–31); these are nuances that are currently masked when measuring clearance curves using parasitemia estimated by methods such as qPCR (32).

It is critical that parasite clearance rates with ACT regimens are assessed routinely across malaria endemic settings. Our objective was to determine the variation in ACT clearance over 72 h among parasite clones found in children with acute uncomplicated *P. falciparum* malaria from Kenya and Tanzania, and to assess risk factors for prolonged parasite clearance times. Participants were followed longitudinally, at 0, 24, 48, and 72 h after administration of artemether-lumefantrine (AL), and subsequently weekly for 28 days. Kenya and Tanzania are countries in which persistence of parasitemia at 72 h is common (19, 20), and we make use of a novel framework for detecting artemisinin susceptibility *in vivo* that can detect low frequency resistant parasites in humans. We also describe risk factors associated with slower clearance rates, including host, environmental, and parasite genetic factors, and show that parasite clearance rates appear to be primarily driven by parasite genetics rather than other clinical characteristics. Early detection, surveillance, and containment are all critical to inform the potential for artemisinin resistance emergence in Africa and to identify the genetic and ecological mechanisms which could enhance spread.

## Materials and Methods

### Study Population

One hundred and fifty children aged 2–10 years who presented with acute uncomplicated *P. falciparum* malaria infection were enrolled in an observational study at each of two sites: Ahero, Kisumu District, Kenya, and Yombo/Fukayosi, Bagamoyo District, Tanzania, between September 2016 and July 2018. At screening, participants completed a clinical, behavioral, and environmental questionnaire measuring characteristics including age, sex, previous malaria episodes, and previous antimalarial use. Participants were eligible to participate if they had a minimum of 500 parasites per 200 white blood cells as measured by microscopy. Study participants were treated with weight-based dosing of AL according to national guidelines with directly observed therapy. Blood was collected at time 0 (the time of the first dose), and at 24, 48, and 72 h after receiving AL. Serum samples for serology were collected at Day 0 and serum samples for pharmacokinetic analysis of lumefantrine were collected at Day 7. All participants were followed weekly for 28 days for evaluation of recurrent parasitemia by microscopy. Informed consent was obtained from a parent or legal guardian and age-appropriate assent was obtained. The study was approved by Institutional Review Boards at the University of North Carolina at Chapel Hill, the Kenya Medical Research Institute and the Muhimbili University of Health and Allied Sciences.

### Determination of Parasitemia

DNA was extracted from 200 μL of whole blood sampled from each participant at each timepoint using Qiagen QiaAMP DNA extraction kits (Qiagen, Hilden, Germany). The extracted DNA was suspended in an equal volume of elution buffer. DNA was quantified using a real-time PCR assay for *P. falciparum* lactate dehydrogenase (*pfldh*) (33) from samples at 0-, 24-, 48-, and 72-h timepoints. As controls, DNA was extracted from mocked clinical samples using cultured parasites (3D7, MRA-102, BEI Resources, Manassas, VA) and human whole blood at known concentrations. Using this standard curve, parasitemia was determined for each sample. All PCRs were performed in duplicate and required both replicates to be positive.

### Parasite Genotyping

To determine reinfection or recrudescence, WHO recommended genotyping of *msp2, msp1*, and *glurp* were conducted as previously described for pairs of initial and recurrent parasitemia (34). Genotyping for reinfection and recrudescence followed the original sequential approach proposed by the World Health Organization (WHO), genotyping *msp2* and *msp1*, using previously published methods (34–36). PCR fragments were sized by gel electrophoresis and called by two investigators using previous published cutoffs (35). Stocks of genomic DNA from Biodefense and Emerging Infections Research Resources Repository (BEI Resources)/ Malaria Research and Reference Reagent Resource Center (MR4) were used as positive controls.

Amplicon deep sequencing of Apical Membrane Antigen 1 (*ama1*) from samples over the first 72 h was done using a PCR based indexing strategy (Supplementary Tables S1, S2). All samples were amplified in technical duplicates. A target specific amplification was initially carried out, followed by 0.8X Ampure bead cleaning and a second PCR for Illumina barcode/sequencing adapter addition (Supplementary Tables S3, S4). Controls of known mixtures containing 4 strains (7G8, HB3, DD2, and 3D7) were included in duplicate at a concentration of 4 parasites/μL and 16 parasites/μL on all amplification plates. Amplicons were quantified and pooled in equimolar proportions. Pools were cleaned with 0.65X Ampure beads and eluted in a low-EDTA TE buffer. Final libraries were sequenced using 2X150 bp chemistry on Illumina MiSeq at the Rhode Island Genomics and Sequencing Center. Sample specific reads were demultiplexed and quality assessed using a sliding window average threshold (sliding window = 50 bp, step size = 5 bp, quality threshold = 20). The reads were processed using default Illumina settings of SeekDeep v3.0.0 and collapsed to individual haplotypes (37). Therefore, each haplotype represented a unique sequence of *pfama* within the amplified product of the gene (32). Additional information on haplotype calling is provided in the Supplementary Material. Individual samples with reads <250 or a sum of replicate reads <250 were excluded. Haplotypes representing >0.5% within sample frequency, supported by at least 10 reads and occurring in both PCR replicates were included (37). Complexity of Infection (COI) was determined by the number of infecting haplotypes at a timepoint.

Given the association of the N86Y mutation in *Plasmodium falciparum* multi-drug resistance gene 1 (*pfmdr*1) with susceptibility to lumefantrine (38), we sequenced this polymorphism from samples with available DNA. We used previously described primers to conduct conventional PCR and Sanger sequenced the product using either the forward or reverse primer at Eton Bioscience (High Point, NC) and Genewiz (Morrisville, NC) (39). PCR was conducted using HotStarTaq Master Mix (Qiagen, Hilden, Germany), 500 nM of each primer and 2.5 μl of template DNA in 25 μl volume. PCR involved 95°C for 15 min, followed by 35 cycles of 94°C for 30 s, 50°C of 1 min and 72°C for 1 min and a final 10 min 72°C final extension. Data was analyzed using Geneious Prime (San Diego, CA).

### Serology

To assess prior exposure to *P. falciparum* malaria infection, IgG antibodies against Merozoite Surface Protein 1 (MSP1) and Apical Membrane Antigen 1 (AMA1) were determined using multiplexed Luminex assay (40). MSP1 and AMA1 antigens were coupled to Bio-Plex COOH carboxylated non-magnetic beads (1.25 × 10^7^) at 100 g/500 μL according to the manufacturer's protocol. Raw mean fluorescence intensities (MFIs) representing IgG antibody levels were measured using BioPlex 200 Multianalyte Analyzer (Bio-Rad Laboratories) from a minimum of 50 beads for each analyte, with positive and negative controls included in each plate (40). To remove non-specific signal, MFI from BSA-beads from each participant was subtracted to raw MSP1 and AMA1 MFI values, representing specific IgG antibody levels. Average MFI values in bead-only negative control wells were used to normalize the median fluorescence intensity of each individual sample to derive final MFI.

### Lumefantrine Drug Levels

Plasma collected on Day 7 was tested at the Center for Research in Therapeutic Sciences (CREATES, Nairobi) for lumefantrine levels using HPLC-MS/MS (41). Samples were analyzed in a singlicate. The lower limit of detection for the method was 5 ng/ml.

### Host Genotyping

In order to assess the impact of known polymorphisms that affect malaria susceptibility on molecular persistence, nine mutations in five genes [CD36 T1264G, G6PD med, G6PD +376, G6PD +202, HbS, HbC, HbE, Duffy (null), and Blood Group O] were targeted for molecular inversion probe (MIP) designs using MIPTools (42). Eight of these polymorphisms have previously been associated with decreased susceptibility to or decreased disease from falciparum malaria (43–47). One (Duffy null) is associated with resistance to vivax infection and was included as a control due to its high expected prevalence in the population. A total of 14 MIPs were designed (Supplementary Table S5). Probe sequences and additional genomic information is provided in the Supplementary Material. MIP captures and data analyses were carried out as described for previous human genetic analysis (48). MIP libraries were sequenced on the Brown Genomics Core Illumina NextSeq (Providence, RI).

### Data Analysis

Our primary outcome was prolonged parasitemia clearance times, as measured by (A) presence of PCR detectable PCR parasitemia at 72 h and (B) the slope of the relationship between log_*e*_ parasitemia and time (49), based on evidence that parasite clearance follows a linear relationship on the log scale (50).

Risk factors included study site, sex, age, socioeconomic status, recent antimalarial use, presence of a water source within a 2-min walk from the home, bed net use, the number of mosquito nets within the home, MSP1 and AMA1 serology results, AL drug Concentrations on day 7, parasitemia and complexity of infection (COI) at enrollment (0 h), and human host genotypes (25–28). Age was calculated as a continuous variable in years. An asset-based approach, using the values from the first principal component of a principal components analysis, was used to calculate socioeconomic status wealth quartiles (51). Variables used for this metric included housing characteristics (e.g., roof and wall materials), water source, and ownership of household appliances (e.g., televisions and radios).

We modeled the relationship between risk factors and prolonged parasitemia clearance times with generalized linear models, calculating odds ratios for the presence of detectable parasitemia at 72 h as a binary variable (presence/absence). Haplotype-specific estimated parasite clearance slopes were also calculated by fitting linear models to the decline in log_*e*_ total parasite density. Estimates were calculated for those variants which were detected at a minimum of 3 time points within each individual. All tabulations, figures, and models were run using R 3.6.2 (R Foundation for Statistical Computing, Vienna, Austria).

## Results

### Participant Characteristics

Baseline demographic and serology data were available for 150 participants at each site (Table 1). In the overall population, 137 (45.7%) participants were female, and the mean age was 6.3 years (SD: 2.6). Most participants (146, 97.3%) from Kenya lived within a 2-min walk from a water source, but only 43 (28.7%) participants from Tanzania reported a water source nearby. Nearly all participants (96.0%) were from households that had mosquito nets for sleeping, with a mean of 2.6 nets (SD: 1.2) per household. Only 5 (1.7%) participants reported using an antimalarial in the last 28 days.

**Table 1:** Baseline characteristics of study participants by study site.

**Type**	**Variable**	**Kenya (*n* = 150)**	**Tanzania (*n* = 150)**	**Overall (*n* = 300)**	
Demography	Female sex	74 (49.3)	63 (42.0)	137 (45.7)	*n* (%)
	Age (years)	5.1 (2.2)	7.5 (2.4)	6.3 (2.6)	Mean (SD)
	Water within a 2 min walk from the house	146 (97.3)	43 (28.7)	189 (63.0)	*n* (%)
	Household has mosquito nets	142 (94.7)	146 (97.3)	288 (96.0)	*n* (%)
	Number of mosquito nets	2.3 (1.0)	3.0 (1.2)	2.6 (1.2)	Mean (SD)
	Antimalarial use in the last 28 days	3 (2.0)	2 (1.3)	5 (1.7)	*n* (%)
Serology	AMA1 antibody level (MFI)	10,908 (5,935)	10,465 (5,419)	10,699 (5,692)	Mean (SD)
	MSP1 antibody level (MFI)	7,009 (3,808)	6,301 (3,793)	6,675 (3,811)	Mean (SD)
PCR	qPCR parasitemia (parasites/μL)	70,168 (78,971)	58,675 (58,466)	64,922 (70,467)	Mean (SD)
	Complexity of infection (# haplotypes)	2.0 (1.3)	1.6 (1.0)	1.8 (1.2)	Mean (SD)

*Additional missing values: age (KE = 2, TZ = 4), bednet use (KE = 1, TZ = 2), # of bednets (KE = 8, TZ = 4), antimalarial use (KE = 1, TZ = 2), AMA1 (KE = 2, TZ = 18), MSP1 (KE = 2, TZ = 18), COI (KE = 12, TZ = 66), parasitemia (KE = 0, TZ = 24)*.

Multiple host factors relating to malaria exposure, malaria susceptibility, and drug levels were measured. Previous exposure to malaria was high at both sites with a mean MFI for anti-AMA1 and anti-MSP1 antibodies of 10,699 (SD: 5,692) and 6,675 (SD: 3,811), respectively (Supplementary Figure S1). The frequency of human genotype mutations was similar by site (Supplementary Figure S2): 19.0% (22/116) of patients were heterozygous for CD36 T1264G, 15.6% (17/109) were heterozygous for G6PD +202, 2.8% (3/109) were homozygous for G6PD +202, 24.6% (50/203) were heterozygous for G6PD +376, and 23.2% (47/203) were homozygous for G6PD +376. Only 9.3% (20/214) of patients were homozygous for blood group O and 9.5% (20/210) were heterozygous for HbS. No participants from either site had G6PD med, HbC, or HbE mutations. All participants were homozygous for the Duffy antigen. AL drug levels at day 7 were detected with a mean value of 184.09 ng/mL (SD = 254.05 ng/mL, *n* = 122) in Kenya and 529.46 ng/mL (SD = 368.95 ng/mL, *n* = 47) in Tanzania. Fourteen patients from Kenya had no drug detected on day 7.

### Parasite Clearance by qPCR

**Figure 1:**
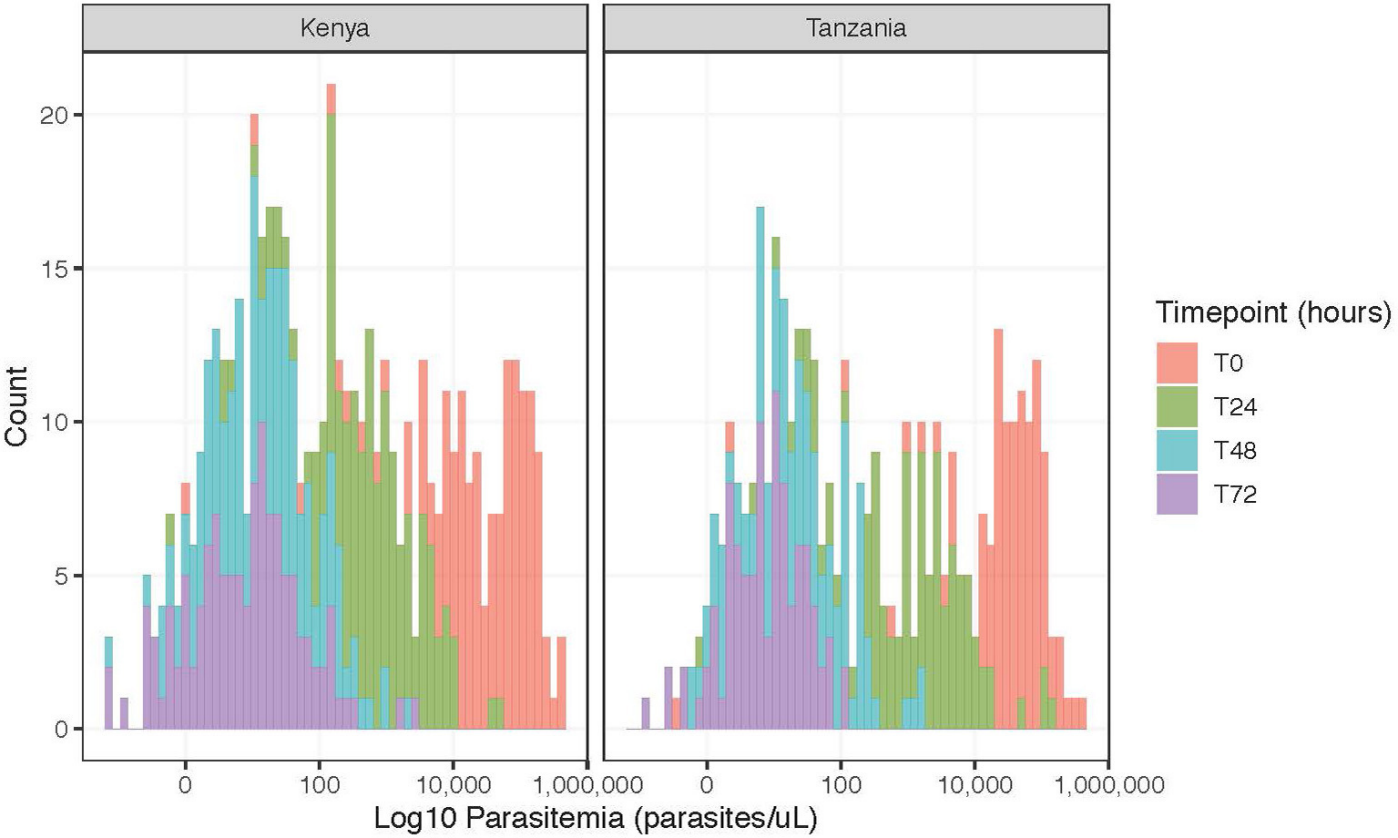
Frequency of log parasitemia counts (parasites/μL) for participants with complete data (Kenya, *n* = 142; Tanzania, *n* = 100), stratified by time point.

The mean parasitemia by qPCR at enrollment was 64,922 parasites/μL with a range from 0.34 to 407,645 parasites/uL (Figure 1). Only five individuals presented with parasitemias <50 parasites/μL based on qPCR. After treatment, the parasitemia distribution curve overtime shifted toward zero (Figure 1), with an average percent change of 99.5% in Kenya and 91.3% in Tanzania from enrollment to 72 h among participants with complete data at all timepoints (Kenya: *n* = 142, Tanzania: *n* = 100) (Supplementary Table S6). Despite decreasing parasitemia across nearly all participants at all time points (Supplementary Figure S3), 121 (85.2%, 121/145 with qPCR data at 72 h) participants from Kenya and 96 (96.0%, 96/100 with qPCR data at 72 h) participants from Tanzania still had qPCR-detectable parasitemia at 72 h. No participants had microscopy detectable parasitemia at 48 or 72 h.

### AMA Genotyping

There were 63 unique haplotypes detected in our study population, 51 detected in Kenya, and 44 in Tanzania. Haplotype frequency varied over time (Supplementary Table S7). The number of reads on average were, 10,394 (SD: 5,256) reads at 0 h, 5,961 (SD: 6,868) reads at 24 h, 3,289 (SD: 7,400) reads at 48 h, and 2,052 (SD: 5,290) reads at 72 h (Supplementary Table S8). The mean COI was 1.8 haplotypes (SD: 1.2) with a range from 1 to 8 (Supplementary Figure S4). We amplified and sequenced 8 replicates of controls using an estimated 90:10 two-strain mixture, finding low error around the estimated frequency, with means of 0.89 (SD: 0.006) and 0.11 (SD:0.006).

### Pfmdr1 Genotyping

In total, DNA was available from 150 Kenyan participants and 130 Tanzanian participants for genotyping. The success rate of sequencing was 64% (96/150) in Kenya and 80.8% (105/130) in Tanzania. All samples contained the N86 (wild type) allele associated with increased tolerance to lumefantrine. Theses alleles were found in individuals with and without molecular persistence at 72 h. Given no 86Y (mutant) alleles were found, risk could not be determined.

### Risk Factor Assessment

Participants from Tanzania had 4.17 (95% CI: 1.52 to 14.64, *p* = 0.01) times the odds of having detectable parasitemia at 72 h post-treatment compared to participants from Kenya (Table 2). Risk factors include higher parasitemia at baseline with an odds ratio (OR) of 1.02 (95% CI: 1.01–1.03, *p* = 0.01) for every 1,000 parasites/μL increase. COI at baseline was protective against detectable parasitemia at 72 h with an OR of 0.70 (95% CI: 0.53–0.94, *p* = 0.01) for every additional haplotype in an infection. None of the other measured demographic, serological, or human host genetic risk factors displayed any association with detectable parasitemia at 72 h, although precision was low for human genetic factors due to the small sample size.

**Table 2:** Bivariate associations between demographic and biological risk factors and detectable parasitemia at 72 h (*N* = number of participants).

**Type**	**Variable**	***N***	**OR**	**95% CI**		***p*-value**
Demography	**Site** (TZ vs. KE)	242	4.17	1.52	14.64	0.01
	**Female sex**	242	1.41	0.61	3.36	0.4
	**Age** (years)	239	1.02	0.86	1.20	0.9
	**Wealth quartiles**	238	0.77	0.51	1.12	0.2
	**Water within a 2 min walk from the house**	242	0.38	0.11	1.03	0.08
	**Household has mosquito nets**	241	1.46	0.08	9.04	0.7
	**Number of mosquito nets**	234	1.29	0.87	2.02	0.2
	**Antimalarial use in the last 28 days**	240	1.03	0.96	-	0.8
Serology	**AMA1 antibody level** (1,000 unit change)	237	1.00	0.93	1.07	1.0
	**MSP1 antibody level**(1,000 unit change)	237	0.97	0.87	1.09	0.6
PCR	**qPCR parasitemia at baseline** (1,000 parasites/uL change)	242	1.02	1.01	1.03	0.01
	**Complexity of infection** (# haplotypes)	222	0.70	0.53	0.94	0.01
AL levels	**AL levels at day 7** (100 ng/mL change)	99	0.92	0.77	1.14	0.4
Host genotyping	**CD36 T1264G** (heterozygous)	90	3.54	0.63	66.86	0.2
	**G6PD + 202** (heterozygous)	84	1.03	0.15	20.63	1.0
	**G6PD + 376** (heterozygous)	165	1.05	0.26	5.18	0.9
	**G6PD + 376** (homozygous)	165	0.49	0.14	1.82	0.3
	**Blood group O** (homozygous)	175	1.71	0.29	32.69	0.6
	**HbS** (heterozygous)	170	1.27	0.22	23.88	0.8

*TZ, Tanzania; KE, Kenya*.

### Haplotype-Specific Slopes

**Figure 2:**
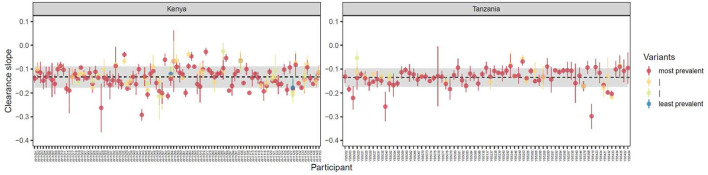
Variant-specific estimated clearance slopes of parasites isolated from individuals in Kenya (*n* = 99) and Tanzania (*n* = 71) ± 1 standard error. Clearance slopes were estimated by fitting linear models to the decline in log_e_ total parasite density. Each point represents a single study participant. Estimates are shown only for those variants which were detected at a minimum of 3 time points. Dashed horizontal lines indicate the mean estimated clearance slope of parasites (defined as the average slope by qPCR) in each region ± 1 standard deviation (shaded regions).

Haplotype specific slopes were determined for all haplotypes that were detected at a minimum of three time points as previously described (32, 52). The mean estimated clearance slopes of parasites, based on total parasite densities by qPCR, isolated from Kenya and Tanzania were −0.13 (SD: 0.04) and −0.14 (SD: 0.04), respectively, with most haplotype-specific slopes hovering close to the mean (Figure 2). Within-individual level haplotypes are shown in Supplementary Figure S5. Within each country, specific haplotypes were not associated with lower slope.

### Reinfection and Recrudescence

A total of 45 individuals had recurrent parasitemia during the 28-day follow-up (Kenya = 33 and Tanzania = 12). Samples were deemed a re-infection if pairs contained unique bands at one or both genes. Band sharing at a single site (with failure at the second site) was deemed uninterpretable. Recrudescence was defined as band sharing at both sites. *glurp* was not genotyped due to its poor discriminatory power. We were able to successfully genotype 39 (87%) of these sample pairs as reinfection or recrudescence (32 from Kenya and 7 from Tanzania). In total, 6 infections were classified as recrudescence by having matching alleles in both loci (3 in Kenya and 3 in Tanzania). All other samples had novel alleles in at least one assay. A total of 2 samples (one in each country) were deemed indeterminate based on a single site amplifying. The remaining 5 samples had no bands generated. There was no association between recurrent parasitemia and persistence of PCR positivity at 72 h (*p* = 0.9) or COI at enrollment (*p* = 0.9).

## Discussion

The majority of participants at both of our study sites had detectable *P. falciparum* parasitemia by PCR at 72 h after treatment initiation with AL. No patients had detectable infection by microscopy at 48 or 72 h. High parasitemia and low COI at baseline were the only factors associated with detectable parasitemia at 72 h. Haplotype-specific estimated parasite clearance slopes were grouped around the mean with no association detected between specific haplotypes and slow clearance rates, with the majority of infections being monoclonal at all timepoints. Few recrudescences were observed after treatment using *msp1/msp2* genotyping. Neither country has documented widespread presence of validated K13 mutations (53, 54).

The clinical significance of persistent submicroscopic parasitemia after ACT therapy remains uncertain (55). Sustained PCR-detectable parasitemia at day 3 has been associated with recurrent microscopic parasitemia, longer gametocyte carriage duration, and a higher likelihood of infecting mosquitoes (20). Multiple studies have shown persistence of DNA for well beyond 3 days post-treatment with ACT, including among travelers with no chance of reinfection (56, 57). It has been shown that these parasites remain transcriptionally active suggesting continued viability (58, 59). A recent study has suggested that ring stage density determined by transcript specific qRT-PCR of a ring specific transcript is associated with recurrent parasitemia at Day 42 (59). While these findings are intriguing given a recent report of failure associated with prolonged PCR detected parasitemia (21), further research of persistent detection of malaria post ACT therapy is needed to determine clinical significance.

A recent review has shown that PCR positivity at day 3 is variable and becoming more frequent in sub-Saharan Africa (55). Most participants in our study had PCR-detectable parasitemia at 72 h; 85.2% of patients in Kenya, and 96.0% of patients in Tanzania. These estimates are higher than previously published results from one of our study sites in Tanzania, showing that 43.8% of patients had PCR-detected parasitemia 72 h after administration of AL (28), and higher than estimates from Kenya where 33.3% of children had qPCR-detectable parasitemia on day 3 after treatment with AL (20). The temporal patterns of prevalence of day 3 PCR positivity are also variable. Studies in Angola have shown a persistent increase in the rate of day 3 parasitemia, while studies in Tanzania have suggested variation over the years (28, 60).

Previous work has shown that persistence of submicroscopic day 3 parasitemia is associated with multiple clinical and demographic factors, including pre-treatment parasitemia, anemia, younger age (<5) and fever at baseline (20, 28). We also see that high parasitemia at baseline impacts clearance, as expected (61), but the COI was inversely related, with participants with low COI being more likely to have detectable day 3 parasitemia. We postulate that infections with a lower COI are caused by haplotypes for which the individual does not have pre-existing strain specific immunity. In the absence of treatment, these strains have a large within-host advantage, leading to their dominance and possible competitive exclusion of other strains, resulting in a lower COI. In the presence of treatment, this lack of host immunity could contribute to longer parasite clearance times. Immunity has been routinely linked to antimalarial efficacy (54, 62–65). While our population appears highly exposed to malaria based upon our serologic profiles, these assays do not provide any information on the strain-specificity of that immunity. Given the use of a single locus for genotyping, we did not identify any specific parasite haplotype associated with persistent parasitemia in this study. Other factors, including host genetic polymorphism and environmental context were not associated with a difference in risk for day 3 positivity.

This study has multiple strengths including the use of 2 independent sites using the same protocols contemporaneously, a controlled environment with routine sampling, and the use of genotyping tools to determine human genotypes and haplotype specific clearance. Given the growing concerns about the efficacy of artemisinin-based combination therapies in sub-Saharan Africa, these data add to our current understanding of parasite clearance, the clinical metric of resistance. There are, however, multiple limitations to the study. First, we did not sample between 0 and 24 h and therefore cannot calculate slope with a lag time. Second, we were also limited by small sample size for multiple analyses, especially for day 7 drug levels and human genotyping, due to extraction failure or missingness. Lastly, we are reliant on a single locus for our haplotype-specific clearance genotyping, which limits the ability to truly define strains (52). Multi-locus genotyping would potentially be more powerful but would be significantly hampered by the low parasitemia levels in samples taken late during therapy (42, 64).

Given the preliminary and concerning finding of artemisinin resistance in sub-Saharan Africa, vigilance is needed in the continued evaluation of emergence and spread. There remains uncertainty about the clinical implication of day 3 (or later) submicroscopic or PCR detectable parasitemia on clinical outcomes. Therefore, programs should continue to monitor for day 3 microscopic persistence, genotypically corrected recrudescence and molecular markers for the evaluation of the emergence of artemisinin resistance in sub-Saharan Africa. The within-host diversity data from this and other studies have the potential to help us understand how the resistance may spread once it evolves. It may provide insight into how strain-specific immunity can impact ACT therapy. It can also be used to better understand relative fitness of drug resistant parasites and within host competition that may impact spread after emergence. Studies that are monitoring ACT efficacy should consider including COI-specific analyses and day 3 microscopic persistence, especially early-on as resistance emerges.

## Data Availability Statement

The original contributions presented in the study are publicly available. This data can be found here: SRA under the Bioproject ID (PRJNA814743) and https://github.com/IDEELResearch/Pf-COI-in-KE-TZ.

## Ethics Statement

The studies involving human participants were reviewed and approved by University of North Carolina at Chapel Hill. Written informed consent to participate in this study was provided by the participants' legal guardian/next of kin.

## Author Contributions

BN, AMM, JO, and JJ were responsible for study design. BN, LM, BOg, AMM, CF, BOd, HA, and JO collected samples. OK and MD performed PCR. AMM, OA, JB, CF, and PO conducted serological and genetic analysis. KM, BOd, MD, and HT performed data cleaning. NM, AR, HT, KM, and MD-F conducted data analysis. HT, KM, and JJ drafted the initial manuscript. All authors read and approved the final manuscript.

## Funding

This project was funded by the National Institutes of Health (R01AI121588 and K24AI134990 to JJ). The funding body had no role in the design of the study and collection, analysis, interpretation of data, or in writing the manuscript.

## Conflict of Interest

The authors declare that the research was conducted in the absence of any commercial or financial relationships that could be construed as a potential conflict of interest.

## Publisher's Note

All claims expressed in this article are solely those of the authors and do not necessarily represent those of their affiliated organizations, or those of the publisher, the editors and the reviewers. Any product that may be evaluated in this article, or claim that may be made by its manufacturer, is not guaranteed or endorsed by the publisher.

## Abbreviations

ACT: artemisinin-based combination therapy
AL: artemether-lumefantrine
AMA1: Apical Membrane Antigen 1
KE: Kenya
MFI: median fluorescence intensity
MSP1: Merozoite Surface Protein 1
OR: odds ratio
TZ: Tanzania
WHO: World Health Organization.
