# Supplementary material for: Low Complexity of Infection Is Associated With Molecular Persistence of Plasmodium falciparum in Kenya and Tanzania
Source: Front Epidemiol. 2022 Jun 6;2:852237. doi: 10.3389/fepid.2022.852237 (PMC10910917; doi:10.3389/fepid.2022.852237)
Supplement: Supplementary file 4 [file Data_Sheet_2.PDF]

SUPPLEMENTARY TABLES AND FIGURES

Low Complexity of Infection is Associated with Molecular Persistence of *Plasmodium falciparum* in Kenya and Tanzania

Hillary M. Topazian[1], Kara A. Moser[2], Billy Ngasala[3], Peter O. Oluoch[4,5], Catherine S. Forconi[4], Lwidiko E. Mhamilawa[3,6], Ozkan Aydemir[7], Oksana Kharabora[2], Molly Deutsch-Feldman[8], Andrew F. Read[9], Madeline Denton[2], Antonio Lorenzo[10], Nicole Mideo[10], Bernhards Ogutu[5], Ann M. Moormann[4], Andreas Mårtensson[6], Boaz Odwar[5], Jeffrey A. Bailey[7], Hoseah Akala[5], John Michael Ong'echa[5], Jonathan J. Juliano[1,8,11,12*]

[1] Imperial College, London, UK
[2] Institute for Global Health and Infectious Diseases, University of North Carolina, Chapel Hill, NC, USA 27599
[3] Muhimbili University of Health and Allied Sciences, Dar es Salaam, Tanzania
[4] Department of Medicine, University of Massachusetts Chan Medical School, Worcester, MA, USA 01605
[5] Center for Global Health Research, Kenyan Medical Research Institute, Kisumu, Kenya
[6] Department of Women's and Children's Health, International Maternal and Child Health, Uppsala University, Uppsala, Sweden
[7] Department of Pathology and Laboratory Medicine, Brown University, RI, USA, 02906
[8] Department of Epidemiology, Gillings School of Global Public Health, Chapel Hill, NC, USA 27599
[9] Department of Entomology, Penn State University, University Park, PA, USA 16802
[10] Ecology and Evolutionary Biology, University of Toronto, Ontario, Canada
[11] Division of Infectious Diseases, School of Medicine, University of North Carolina, Chapel Hill, NC, USA 27599
[12] Curriculum in Genetics and Molecular Biology, School of Medicine, University of North Carolina, Chapel Hill, NC, USA 27599

SUPPLEMENTARY METHODS

SeekDeep analysis pipeline

The reads were processed using default Illumina settings of SeekDeep v3.0.0 and collapsed into individual haplotypes as described in Hathaway et al. (2017). Briefly, reads were demultiplexed and quality filtered using a sliding window average threshold (sliding window= 50bp, step size= 5 bp, quality threshold= 20) using the **extractor** function. The following additional *"—extraExtractorCmds"* were also included to account for the random 5' Ns included in the amplicon primers: *--extraExtractorCmds="--primerWithinStart 10"*. Sample level clustering of sequences into haplotypes was performed by **qluster** based on default Illumina parameters. Finally, haplotypes from the qluster step were processed by **processCluster,** while accounting for PCR technical replicates. Additional filters were including in this step using the following flags: *--extraProcessClusterCmds="--strictErrors*

--excludeCommonlyLowFreqHaplotypes --excludeLowFreqOneOffs --replicateMinTotalReadCutOff 250 --sampleMinTotalReadCutOff 100”. The number of unique haplotypes was compared to generate both individual and population-level complexity of infection (COI). Samples with total read count below 250 or a sum of replicate below 250 were excluded. Haplotypes representing >0.5% within sample frequency, supported by at least 10 reads and occurring in both PCR replicates were included.

Table S1. First Round PCR primer sequences for AMA1 library preparation.

Primer	Sequence (5'→3')
Ama1F	GACTCGCCAAGCTGAAGNNNNNNNNNNCCATCAGGGAAATGTCCAGT
Ama1R	ACGTGTGCTCTTCCGATCTNNNNNNNNNNTTTCCTGCATGTCTTGAACA

[1]Ns represent 10 random nucleotide sequences on the 5' end of primers.

Table S2. Second Round PCR primer sequences for AMA1 library preparation

See uploaded excel file: Table S2. Second Round PCR Primers.xls

Table S3. PCR reaction mix.

Primary PCR

Reagents	Stock concentration	Vol (μL)
Q5 Reaction Buffer (NEBs)	5X	5
Pf AMA1 primer (fw)	10μM	1.25
Pf AMA1 primer (rv)	10μM	1.25
dNTPS	10mM	0.5
Q5 High-Fidelity DNA Polymerase	0.02 U/μL	0.25
Molecular crowders (MMC)	5X	5
Water		6.75
Template		5
**Total**		**25**

Nested PCR

Reagents	Stock concentration	Vol (μL)
Q5 Reaction Buffer (NEBs)	5X	5
In-house primers with barcodes and sequencing adaptors (Fw)		1.25
In-house primers with barcodes and sequencing adaptors (Rev)		1.25
dNTPS	10mM	0.5
Q5 High-Fidelity DNA Polymerase	0.02 U/μL	0.25
Molecular crowders (MMC)	5X	5
Water		6.75
Template	Cleaned PCR1 product	5
**Total**		**25**

Table S4. PCR amplification conditions.

**Primary PCR**			
**Programme**	**Temperature**	**Time**	**Cycles**
Initial denaturation	98°C	30 sec	
Denaturation	98°C	10 sec	10
Annealing	63°C	30 sec	
Elongation	68°C	30 sec	
Final elongation	72°C	2min	
**Nested PCR**			
**Programme**	**Temperature**	**Time**	**Cycles**
Initial denaturation	98°C	30 sec	
Denaturation	98°C	10 sec	20
Annealing	63°C	30 sec	
Elongation	68°C	30 sec	
Final elongation	72°C	2 min	

Table S5. Human Molecular Inversion Probes

See uploaded excel file: Table S5. Human Genotyping MIPs.xls

Table S6. Study participant parasitemia (parasites/μL) at enrollment and at 24-, 48-, and 72-hour follow-up visits. Only participants with complete data at each time-point are included. The percent change in parasitemia and relative parasitemia values are calculated for each individual prior to determining the mean.

Variable	Kenya (n=142)	Tanzania (n=100)
**Parasitemia detected (%)**		
Enrollment (0 Hours)	142 (100)	100 (100)
24 Hours	138 (97.2)	100 (100)
48 Hours	136 (95.8)	98 (98.0)
72 Hours	121 (85.2)	96 (96.0)
**Parasitemia (parasites/μL) mean (SD)**		
Enrollment (0 Hours)	68,292 (78,556)	57,773 (60,338)
24 Hours	2,045 (5,730)	6,612 (20,101)
48 Hours	72 (217)	105 (266)
72 Hours	51 (248)	15 (21)
**Average % Δ in Parasitemia**		
Enrollment (0 Hours)	0%	0%
24 Hours	60.3%	63.1%
48 Hours	99.4%	67.8%
72 Hours	99.5%	91.3%
**Relative Parasitemia**		
Enrollment (0 Hours)	1.00	1.00
24 Hours	0.397	0.369
48 Hours	0.006	0.322
72 Hours	0.005	0.087

Note: missing data at time 0, 24, 48, 72 hours is as follows. Kenya: 0,1,4,7; Tanzania: 23,22,30,40

Table S7. Frequency of haplotypes by time.

	X.PopUID	0 hours	24 hours	48 hours	72 hours	missing
1	pfama1.00	35	31	23	15	-
2	pfama1.01	32	27	17	14	-
3	pfama1.02	30	26	20	13	-
4	pfama1.03	28	22	15	13	-
5	pfama1.04	27	22	21	7	-
6	pfama1.05	22	22	16	10	-
7	pfama1.06	17	19	15	10	-
8	pfama1.07	15	13	15	16	-
9	pfama1.08	18	14	12	11	2
10	pfama1.09	14	13	9	4	1
11	pfama1.10	9	8	13	9	-
12	pfama1.11	11	8	7	13	-
13	pfama1.12	13	11	6	8	-
14	pfama1.13	12	14	8	4	-
15	pfama1.14	11	10	9	3	-
16	pfama1.15	9	8	6	8	-
17	pfama1.16	9	10	8	4	-
18	pfama1.17	3	2	13	5	2
19	pfama1.18	4	6	5	6	-
20	pfama1.19	5	5	4	7	-
21	pfama1.20	5	6	3	6	-
22	pfama1.21	3	3	7	6	-
23	pfama1.22	6	5	4	2	-
24	pfama1.23	5	5	3	3	-
25	pfama1.24	1	1	7	6	-
26	pfama1.25	2	1	5	6	-
27	pfama1.26	3	3	3	2	-
28	pfama1.27	3	4	2	2	-
29	pfama1.28	3	4	2	2	-
30	pfama1.29	5	5	1	-	-
31	pfama1.30	5	3	2	1	-
32	pfama1.31	4	3	2	1	-
33	pfama1.32	4	3	2	1	-
34	pfama1.33	4	2	3	-	-
35	pfama1.34	3	2	2	1	-
36	pfama1.35	4	2	1	1	-
37	pfama1.36	2	2	1	2	-

38	pfama1.37	3	3	1	-	-
39	pfama1.38	3	2	1	1	-
40	pfama1.39	1	3	1	1	-
41	pfama1.40	2	3	-	-	-
42	pfama1.41	2	1	1	1	-
43	pfama1.42	1	1	1	1	-
44	pfama1.43	1	1	1	1	-
45	pfama1.44	1	1	1	1	-
46	pfama1.45	1	1	1	1	-
47	pfama1.46	1	1	1	1	-
48	pfama1.47	1	2	1	-	-
49	pfama1.48	2	1	1	-	-
50	pfama1.49	2	2	-	-	-
51	pfama1.50	1	1	2	-	-
52	pfama1.51	-	-	-	2	2
53	pfama1.52	1	1	1	1	-
54	pfama1.53	1	1	1	-	-
55	pfama1.54	1	1	1	-	-
56	pfama1.55	1	-	1	1	-
57	pfama1.56	-	1	1	1	-
58	pfama1.57	1	1	1	-	-
59	pfama1.58	1	1	-	-	-
60	pfama1.59	1	1	-	-	-
61	pfama1.60	2	-	-	-	-
62	pfama1.61	-	-	1	-	-
63	pfama1.62	-	-	-	1	-

Table S8. Number of reads used per time point of successfully genotyped samples.

Site	Time point	N	Mean	SD	Min	Max
**Overall**	**0 hours**	226	10,394	5,256	288	40,045
	**24 hours**	216	5,961	6,868	278	53,465
	**48 hours**	171	3,289	7,400	252	71,697
	**72 hours**	122	2,052	5,290	250	51,959
	**missing**	2	909	445	594	1,224
**Kenya**	**0 hours**	139	10,316	5,883	288	40,045
	**24 hours**	132	4,960	6,023	278	52,342
	**48 hours**	101	3,503	9,027	252	71,697
	**72 hours**	72	2,375	6,670	251	51,959
**Tanzania**	**0 hours**	87	10,518	4,089	996	19,514
	**24 hours**	84	7,534	7,801	338	53,465
	**48 hours**	70	2,981	4,080	272	22,448
	**72 hours**	50	1,585	2,066	250	9,361

Figure S1. AMA1 and MSP1 serology data for individuals upon enrollment, stratified by site (Kenya n=148, Tanzania n=132). Points represent individual values. The box and whisker plot's vertical line represents the minimum and maximum values, while horizontal bars represent the first quartile, median, and third quartile values.

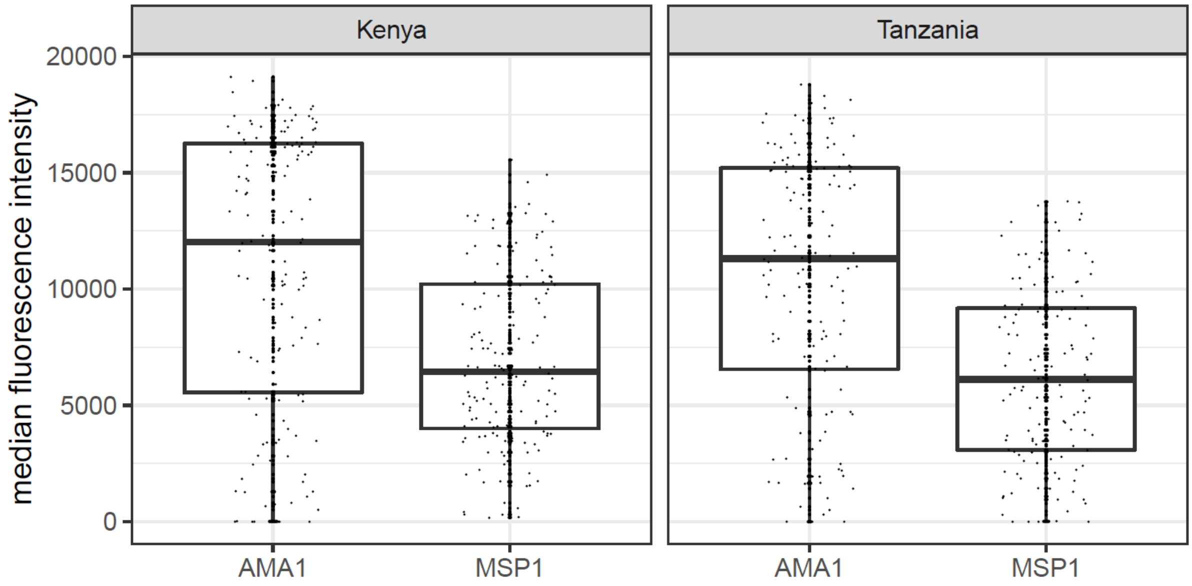

Figure S2. Frequency of human genotype mutations by site.

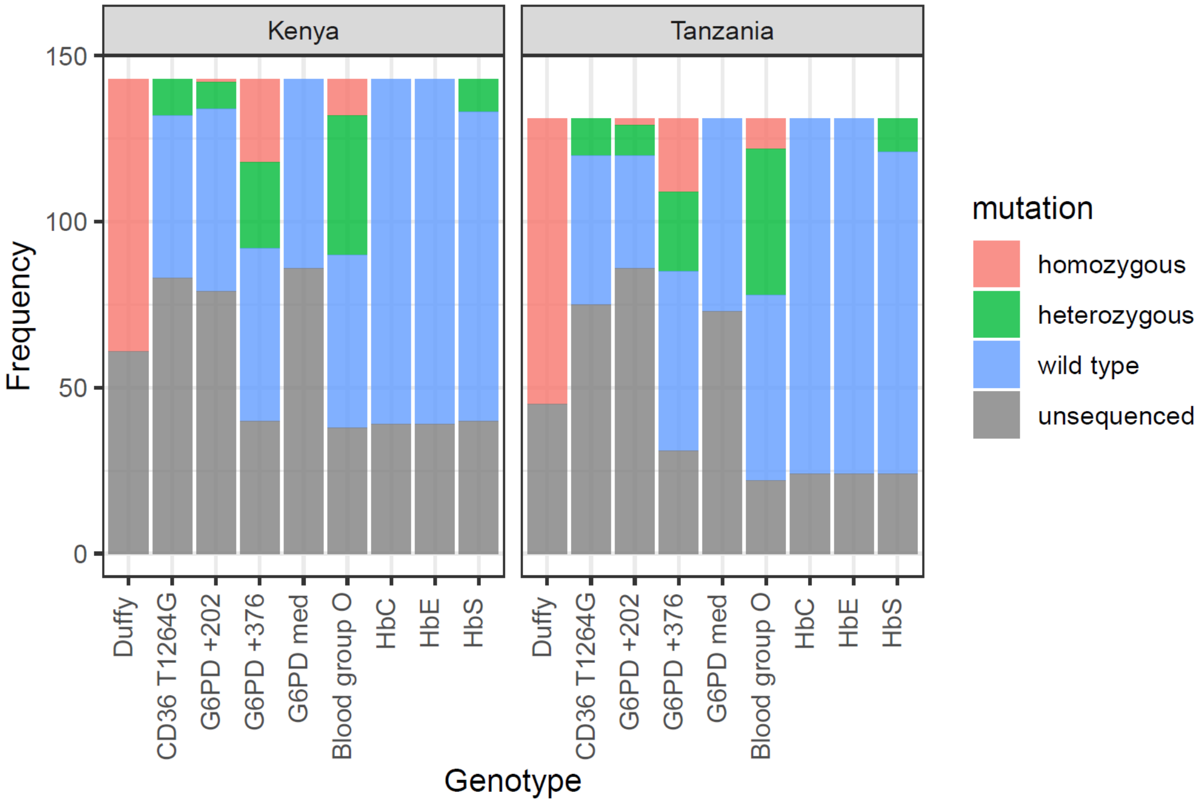

Figure S3. Parasitemia levels (parasites/uL) over time for participants with complete data by time point (Kenya, n=142; Tanzania, n=100). A) Parasitemia per individual. B) Parasite clearance slope per individual, points with 0 parasites/uL were reclassified as 0.01 in order to calculate slope. Bolded colored lines indicate example participants with persistent infections.

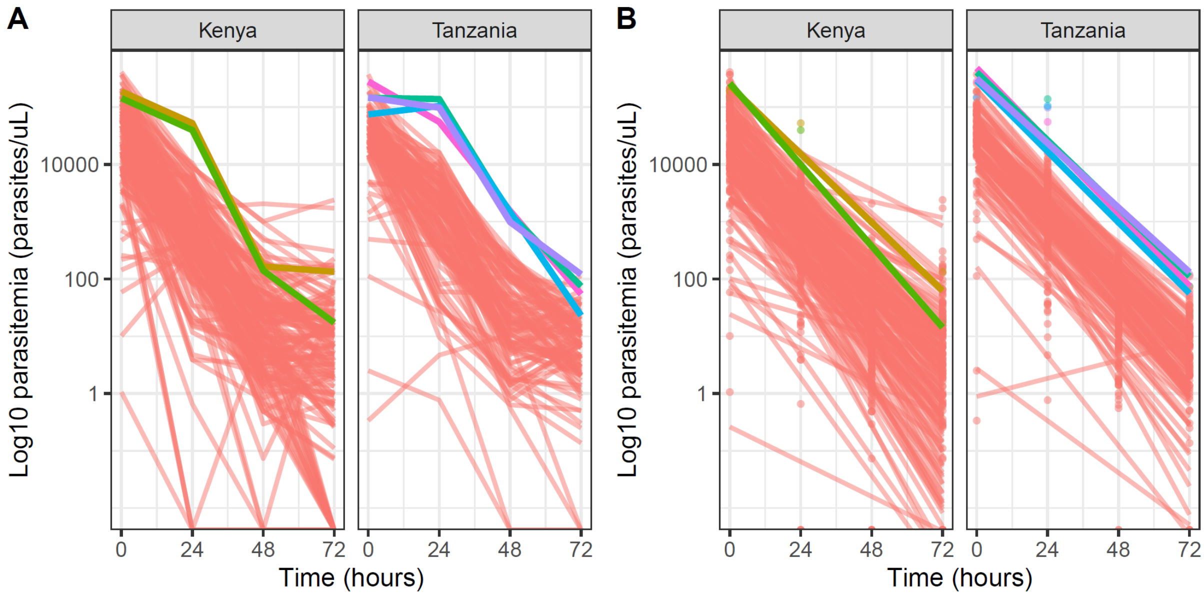

Figure S4. COI distributions for participants at enrollment (Kenya, n=138; Tanzania, n=84).

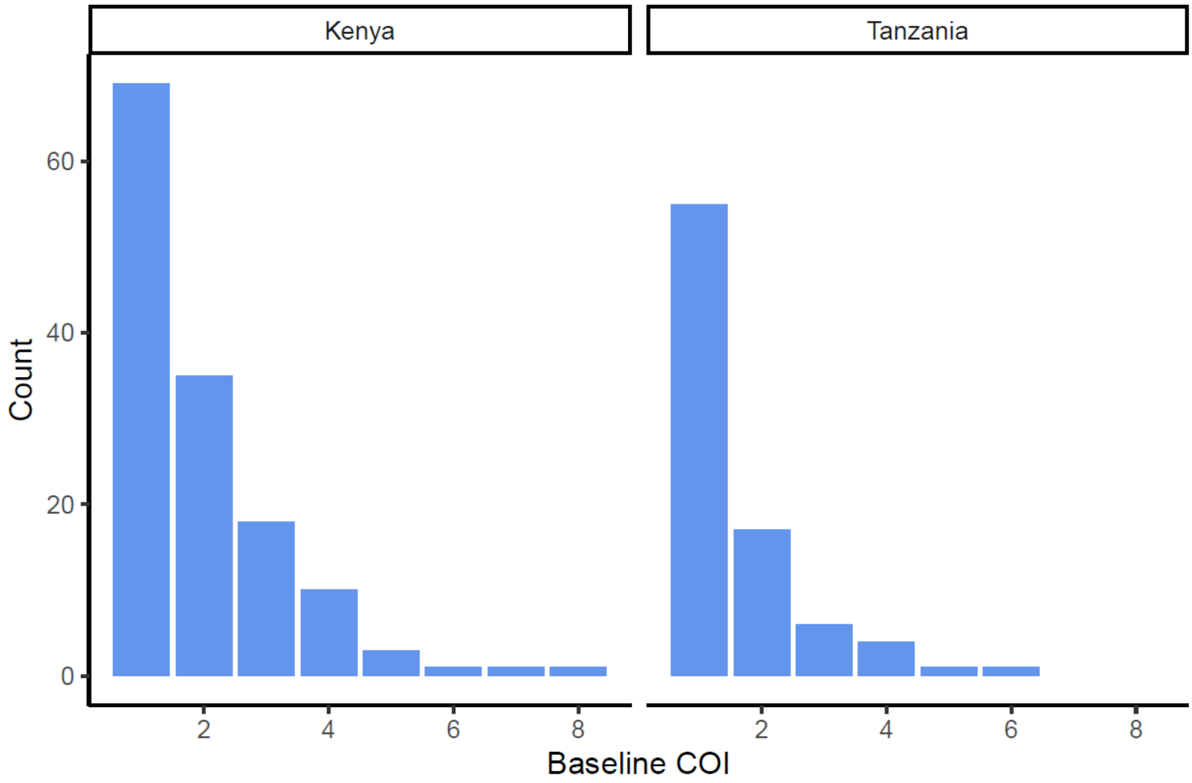

Figure S5. Subpopulation parasite clearance curves from participants in Kenya (n=99) and Tanzania (n=71). Estimates are shown only for those variants which were detected at a minimum of 3 time points. A) Frequency of haplotypes are shown as bar graphs. B) Clearance slopes were estimated by fitting linear models to the decline in $\log_e$ total parasite density. Dashed lines indicate the mean estimated clearance slope of parasites within each individual.

See uploaded file: Figure S5. Clearance Slopes.pdf
