# Supplementary material for: Low Complexity of Infection Is Associated With Molecular Persistence of Plasmodium falciparum in Kenya and Tanzania
Source: Front Epidemiol. 2022 Jun 6;2:852237. doi: 10.3389/fepid.2022.852237 (PMC10910917; doi:10.3389/fepid.2022.852237)

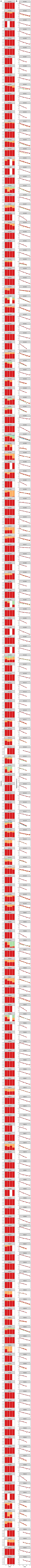

Supplement: Supplementary file 3 [file Data_Sheet_1.PDF]
